# The Prayer Cure

**Published:** 1886-01

**Authors:** 


### THE PRAYER CURE.

Is there any such thing as the prayer cure ? Is there anything in the reports which are constantly coming before the public, of the wonderful cures ? How many times have these questions been asked of us by people who do not believe the stories that are told of them, and by people who believe all that is told them, and only refrain from believing more because more wonderful stories have not reached them. It seems strange indeed that people living in a community where books are easily found to be referred to, and where every means of gaining knowledge of the subject is easily to be attained, do not take pains to inform themselves, and arrive at some fair conclusion upon the subject.

If we had time or space in our present article to go back to India, to Greece and Rome ; indeed all the ancient nations of the East, thousands of years ago, we should find the same class of phenomena, the same kind of facts occurring, and the same, or still more wonderful, things thought of then, than those of modern times. People in those earlier days of the world thought they were the work of some of their numerous gods, the only power they supposed available for such strange phenomena. Many of them worshipped and sacrificed to these strange gods ; but we cannot at this time follow their curious manifestations, and will begin at a period less remote, where the history can be traced and where the transactions can be followed with some degree of assurance.

The "Royal Touch" was commenced during the reign of Charles I, and it was probably just as much a mystery to him as to those who witnessed it. How or by what means he first found that he possessed the power is not given. In those days of kingcraft, which was only exceeded by the priestcraft then existing, anything which was uncommon, or not understood by people generally, passed for a miracle, and so Charles, for his power of healing, was deemed to have the divine grace actually poured out upon him, as if he had been a poor saint instead of one of the common sinners of his time ; but then he was a king, and was as ignorant of the power by which he cured the "king's evil" as those who were cured by his hand.

We must not imagine that all who came to him were cured, for such was not the fact. It was for curing this the one disease that he was most celebrated, but occasionally the afflicted of others came to him and were benefited. The case is so plain as to lead Mr. Collier, in Ecclesiastical History to say, "to thus dispute a matter of fact, is to go to the excess of skepticism, to deny our senses, and to be incredulous even to ridiculousness." This was said when giving a history of Charles the Second, who, it is said, touched during the twenty years he continued the practice, 92,700 patients—sometimes five or six hundred during one sitting. Of course in that age, with the little knowledge of medicines possessed by the people, it was easily magnified into a religious ceremony having its Lord Bishop and a liturgy prepared specially for it. All this aided in the healing process, but thousands were not healed, and of them we get but little report.

Religious faith was very universal in those days, and very strong. It was a very common belief at the time, that Charles the First was the image of Christ—that Cromwell who killed him and reigned in his stead was the devil, and that Charles the Second was Christ come again. It was said that handkerchiefs dipped in Charles' blood cured many of the nobility. We cannot dispute it, for imagination has cured its tens of thousands, and proved a genuine cure too.

But in time others came to cure people by the same process. Valentine Greatrakes of England was found to be equal to any of the royal healers, and he healed by the same process, of which we have abundant testimony. He was, from all accounts, even more successful than the royal healers, as he should have been, and as much sought after. He was for a long time kept busy at his healing.

These healers of diseases have appeared at various times in England, France and Germany, and we presume in all other countries where people have been much more superstitious than they now are in regard to such cases. We cannot refer to all of them, but give these as an illustration.

Let us come to a later period. Take the case of the Cure D'Ars in France, the founder of D'Ars Providence and many other noble works of charity. Persons affected with disease were surprisingly cured by him, and the Abbe Monnin declares that upwards of 24,000 persons came annually from Germany, Italy, Belgium, and all parts of France—even from England—and that in less than six years this number increased to more than 80,000 a year. This man lived until 1859, and the works which he built up remain as a monument to his great power for good in providing for the poor and healing the sick.

Jacob the Zouave was another of the same class of individuals, who healed at once by his remarkable power, as late as 1866. He was discharged from the army, and became a great healer of the same wonderful kind. He made no claim to any religion or any religious aid in the matter, yet healed by the same means, and was discharged from the army because people learned of his great power and rushed to the camp for him to heal them, until they created great confusion by their presence.

Many years ago, before mesmerism was generally known, we knew a man at Lockport, N. Y., who, some years previous to our acquaintance, had this experience : There was a protracted meeting in a church near his home. His wife was a member, but he was not. One night during the meetings he sat at home, reading, and began to feel uneasy. At length he retired to bed, but not to sleep. He tossed and tumbled for a time, then arose and dressed himself, but he could not remain quiet anywhere. Finally he went over to the church, and there he found them all praying for him. He felt assured of the effect of their prayers and, although a very exemplary man, was so struck with the coincidence that he joined the church. Some years after he became acquainted with mesmerism, when the whole thing was explained to his satisfaction. He saw plainly, in trying experiments, how human minds can effect each other, even at a considerable distance.

We knew another man of the name of Simmons, who was a very zealous Methodist. Whenever a certain preacher, who was a powerful man physically, came there, brother Simmons was always so affected, ("had the power," as they said,) that he would stand up, would become rigid and stiff in every joint, unable to move. He was, of course, a shining light to the people of his church and others. Later in life, he too became acquainted with mesmerism, and was able to perceive the source of his affection.

When quite a young man, we were at a camp-meeting in Wayne County, N. Y., when a young woman "had the power." We immediately took her by the hand, and brought her, by the power of mesmerism, to her normal condition. She, if she still remains on earth, will remember it and how angry she was with me, because, she said, she would have had a good time "if we had let her alone."

Jno. M. Spear, then of Boston, was one day "impressed" that he must get his horse and chaise and go some ten or fifteen miles in the country on a road which was totally unknown to him, for some purpose, he could not tell what. He did not feel inclined to go, but the feeling became so strong, that he finally hitched up his horse and started, riding along in silence, following the direction of his great impulse. At length the horse stopped at a poor looking, lonely house, and he went in. A man was sitting in a chair, afflicted with rheumatism, so as to suffer greatly and was unable to walk. Mr. Spear approached asking him what was the matter, and touched him. Immediately the man felt an electric shock all over him and said: "Who are you; what do you want?" He was alarmed at the effect of Mr. Spear's touch, and Mr. Spear repeated it, when the man was entirely healed, but ordered his benefactor away, as he said he was afraid of him. Mr. Spear has had many cases of that kind.

Dr. J. R. Newton, of Boston, had the same faculty in a very eminent degree. On one occasion a man came twenty or thirty miles in the night for him to go and see if he could cure his infant boy—his only child—whom all the doctors of his neighborhood had seen and given up to die. The man begged of the doctor to go at once home with him. The Dr. stretched out his hand towards the man's residence and said: "Go home; your child is cured." The man could not believe him and wanted him to go, but the Doctor refused, saying: "Your child is well." The father went home and found his child, as the Doctor had told him, entirely well, and the mother, by a comparison of time, said that at the time of the night he was at the Doctor's, the child awoke laughing and cured. This is only one of the numerous cases equally strong and well authenticated that were performed by the Doctor, and such cases are occurring in various parts of the country every day.

Are the wonderful cures by these gifted men at all miraculous or are they a part of a great system with which we have not become fully acquainted, that runs in some way through all human society? Much in many cases is effected by pure imagination. In one case arrangements were made by a physician to have a young bedridden woman prayed for at the several churches on a certain day, at a certain hour. At that hour the lady arose and walked, although the

Doctor forgot his promise to get *all* the churches to pray for her, nevertheless she so understood it, and that idea caused her to make the effort, and she was cured.

Such is a brief history of mind cures. Patients would be cured just as soon by united swearing as praying if they only had the idea that *this* was a means of cure. A large portion of the cures are effected by operating upon the imagination, and large numbers are not cured at all. It is just as much a disease if it is imagination as anything else, and until that imagination is cured by prayer or by some other means that looks mysterious to the patients, they will remain so. In some persons the power to operate in this manner of cure is much greater that in others, and the number is far greater in the prayer cure. They fail wholly and entirely, much more generally, than they would have done one or two centuries ago when the mind was a more willing slave to the voice of great men, or religious devotees.

At one time, the writer of this article was at Rochester, N. Y., on a brief business visit. His wife was at his home in Auburn suffering from a severe cough. While conversing with the family of a friend at Rochester the lady of the house said: "I am going to cure Mrs. C's cough," and immediately went into a kind of trance. When she came out of it she said: "she's cured." Of course but little faith was manifested in the cure, but when I got home my wife told me, with surprise, that her cough left "night before last" and that she had not had any since. Nor did she afterwards have a cough, to any extent, while she lived. It was not, most certainly, our peculiar religious ideas that governed it, for we were not what the most of the world then called orthodox, nor was there the least particle of religion or religious idea about it, but extreme doubt on the subject.

We know, too, that the same woman cured the wife of Hon. Waddy Thompson, of South Carolina, after she had been to Europe to all the greatest physicians of the world and they had utterly failed in giving relief. Mr. and Mrs. Thompson called on this lady merely as friends, with no expectation of having anything done for Mrs. T's useless limb, which had been given up as hopeless after her visit to Europe. She was brought into the house by two men. She went away walking like other folks, but did not know how the cure was performed. It was not on account of any religious influence any more than eating, walking or talking. Mrs. Davis, of Providence, R. I., was wholly cured under precisely similar circumstances. The lady at whose house these cures were performed was not a general curer of disease and was never known to the public as such, but these are the facts, and just as much "mind cure" in her case as in thousands of others.

George Fox, and many of the early Quakers, had the power of healing diseases. They did not know what gave them the power and thought it to be some great gift that had been handed over to them because they were nearer than other people to the right way. Now the Quakers do not have it more than other people. The prayer cure is nothing new. It is as old as history, and the same in different ages of the world. When we learn more about prayer we shall know more about the prayer cure: but we may rest assured that there is nothing mysterious in it and that no religious machinery enters into it any more than into thousands of other things which we have come to understand.

